# Association of ten VEGF family genes with Alzheimer's disease endophenotypes at single cell resolution

**DOI:** 10.1002/alz.14419

**Published:** 2024-12-06

**Authors:** Yiyang Wu, Julia B. Libby, Logan Dumitrescu, Philip L. De Jager, Vilas Menon, Julie A. Schneider, David A. Bennett, Timothy J. Hohman

**Affiliations:** ^1^ Vanderbilt Memory and Alzheimer's Center Vanderbilt University Medical Center Nashville Tennessee USA; ^2^ Vanderbilt Genetics Institute Vanderbilt University Medical Center Nashville Tennessee USA; ^3^ Center for Translational & Computational Neuroimmunology Department of Neurology Columbia University Irving Medical Center New York New York USA; ^4^ Taub Institute for Research on Alzheimer's Disease and Aging Brain Columbia University Irving Medical Center New York New York USA; ^5^ Rush Alzheimer's Disease Center Rush University Medical Center Chicago Illinois USA

**Keywords:** Alzheimer's disease, association analysis, dementia, signaling pathway, single cell, single‐nucleus RNA sequencing, transcriptome, vascular endothelial growth factor

## Abstract

**INTRODUCTION:**

Using a single‐nucleus transcriptome derived from the dorsolateral prefrontal cortex of 424 Religious Orders Study and the Rush Memory and Aging Project (ROS/MAP) participants, we investigated the cell type–specific effect of ten vascular endothelial growth factor (VEGF) genes on Alzheimer's disease (AD) endophenotypes.

**METHODS:**

Negative binomial mixed models were used for differential gene expression and association analysis with AD endophenotypes. VEGF‐associated intercellular communication was also profiled.

**RESULTS:**

Higher microglia *FLT1*, endothelial *FLT4*, and oligodendrocyte *VEGFB* are associated with greater amyloid beta (Aβ) load, whereas higher *VEGFB* expression in inhibitory neurons is associated with lower Aβ load. Higher astrocyte *NRP1* is associated with lower tau density. Higher microglia and endothelial *FLT1* are associated with worse cognition performance. Endothelial and microglial *FLT1* expression was upregulated in clinical AD patients compared to cognitively normal controls. Finally, AD cells showed a significant reduction in VEGF signaling compared to controls.

**DISCUSSION:**

Our results highlight key changes in VEGF receptor expression in endothelial and microglial cells during AD, and the potential protective role of VEGFB in neurons.

**Highlights:**

- The prefrontal cortical expression of *FLT1* and *FLT4* was associated with worse cross‐sectional global cognitive function, longitudinal cognitive trajectories, and more Alzheimer's disease (AD) neuropathology.
- The associations between *FLT1* or *FLT4* and AD endophenotypes appear to be driven by endothelial and microglial cells.
- *VEGFB* expression seems to have opposing effects on the Aβ burden in AD depending on cell types, highlighting its potential protective role in neurons.

## 1 BACKGROUND

**FIGURE 1:**
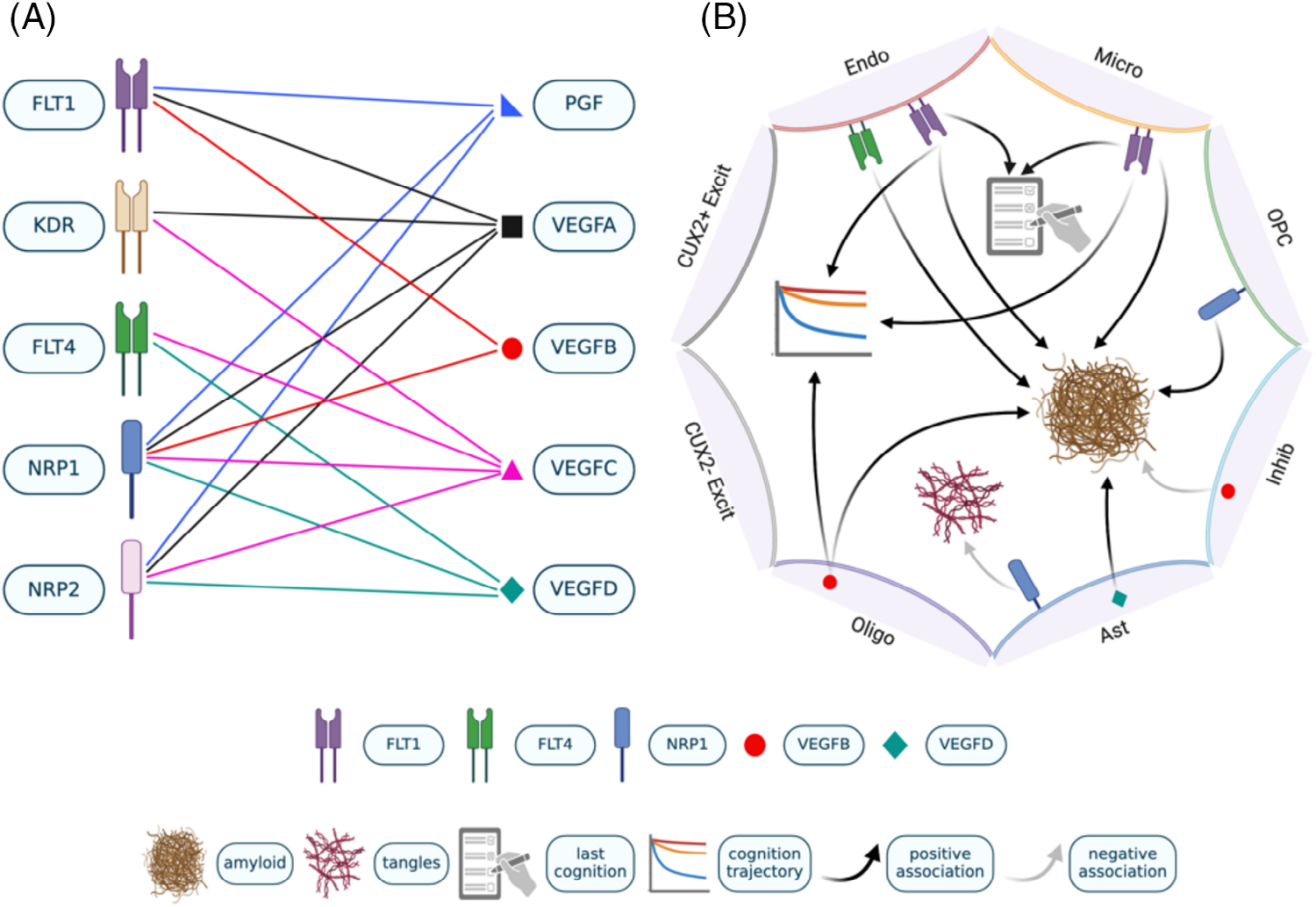
Ten VEGF system and their cell type–specific effect on AD endophenotypes. A, Ten VEGF ligand‐receptor system. B, Association of VEGF and AD endophenotypes in DLPFC brain cells. This figure was created with BioRender.com. AD, Alzheimer's disease; Ast, astrocytes; CUX2– Excit, all excitatory neurons that don't express *CUX2*; CUX2+ Excit, *CUX2*+ excitatory neurons; DLPFC, dorsolateral prefrontal cortex; Endo, endothelial cells; Inhib, inhibitory neurons; Micro, microglia; Oligo, oligodendrocytes; OPC, oligodendrocyte precursor cells; VEGF, vascular endothelial growth factor.

Neurovascular unit dysfunction is a hallmark of the progression of Alzheimer's disease (AD). The vascular endothelial growth factor (VEGF) family of signaling proteins is responsible for the growth and maintenance of cells involved in vascularization and neuralization.^1^ The members of the VEGF family include five ligand genes (*VEGFA*, *VEGFB*, *VEGFC*, *VEGFD*, and *PGF*), three receptor genes (*FLT1*, *KDR*, and *FLT4*), and two co‐receptor genes (*NRP1* and *NRP2*; Figure 1A). These genes play a role in brain injury,^2^ stroke,^3^, ^4^ and the progression of neurodegenerative disease,^5^ particularly AD. Within AD, disruptions in vascularization and circulation of the brain have been shown to contribute to neurodegeneration and the development of neuropathology.^6^, ^7^, ^8^, ^9^, ^10^, ^11^, ^12^, ^13^ As a result, VEGFs have become exciting targets for research to better understand vascular contributions to AD.

The role of VEGFs in AD appears to vary depending on the gene family member. Some family members have protective effects,^9^, ^14^, ^15^ while others contribute to the progression of neurodegeneration.^16^, ^17^, ^18^ Earlier work from our group observed that cerebrospinal fluid levels of VEGFA displayed a protective effect.^15^ When we expanded our analyses to brain tissues we observed that *VEGFB*, *FLT1*, *PGF*, and *FLT4* all appear to contribute to the progression of AD pathology and cognitive decline.^16^ Within the prefrontal cortex, higher expression of these four genes (*VEGFB*, *FLT1*, *PGF*, and *FLT4*) was associated with more rapid cognitive decline and higher pathology burden.^16^ Similar associations are observed when measuring the protein abundance of these VEGF family members in brain tissue and extend to other brain regions including the posterior cingulate gyrus.^18^

To better understand VEGF associations in the human brain within their cellular context, we previously investigated single nucleus expression from 48 *post mortem* dorsolateral prefrontal cortex (DLPFC) samples, evaluating their expression in astrocytes, microglia, oligodendrocytes, oligodendrocyte progenitor cells (OPCs), pericytes, endothelial cells, and excitatory and inhibitory neurons.^18^ Fascinatingly, we found that the robust *FLT1* and *VEGFB* signals that we had observed in bulk tissue were driven by expression in microglia for both genes, along with endothelial cells, oligodendrocytes, and OPCs for *VEGFB*. While results were encouraging, the small sample size and few endothelial and microglial cells in this initial dataset made interpretation challenging.

The goal of this study is to further disentangle the cell type–specific VEGF expression changes in relation to AD endophenotypes by analyzing a much larger AD single‐nucleus RNA sequencing (snRNAseq) cohort (*N* = 424).^19^, ^20^ Specifically, we investigated ten VEGF (*VEGFA*, *VEGFB*, *VEGFC*, *VEGFD*, *PGF*, *FLT1*, *FLT4*, *KDR*, *NRP1*, and *NRP2*) family members’ expression within eight brain cell types to test for differential expression between cognitively normal participants and those with AD, along with expression associations with global cognition (cross‐sectional and longitudinal trajectories) and AD pathology (amyloid beta [Aβ] load and tau neurofibrillary tangle density; Figure 1B). In addition, we evaluated cell type–specific changes in the VEGF‐associated signaling pathway during AD by conducting an intercellular communication analysis for these ten VEGF family members.

## 2 METHODS

### 2.1 Participant characteristics

The participants included in this study were from two longitudinal clinical–pathological cohort studies including the Religious Orders Study and the Rush Memory and Aging Project (ROS/MAP). At enrollment, all participants were free of known dementia and agreed to annual clinical evaluation and the donation of their brain at the time of their death.^21^, ^22^, ^23^ Our analysis was based on 424 post–quality control non‐Latinx White participants of this cohort (Table 1) with an average *post mortem* interval (PMI) of 7.7 hours (standard deviation = 5.1 hours), 68% of whom are females, 52% are clinical AD dementia patients (cogdx = 4 or 5), and 27% carried at least one apolipoprotein E ε4 (*APOE*‐ε4) allele. The cohort has a mean age at death of 89 years old with an average of 13 years of education.

**TABLE 1 alz14419-tbl-0001:** Characteristics of study participants.

	Cognitive normal controls (*N* = 143)	AD patients (*N* = 157)	Total cohort (*N* = 424)	*p*‐value
Age at death (years)	87.20 ± 7.26	91.15 ± 6.56	89.19 ± 6.82	<0.001
Education (years)	16.28 ± 3.45	16.55 ± 3.22	16.31 ± 3.47	0.48
PMI (hours)	7.80 ± 4.99	7.25 ± 4.32	7.71 ± 5.11	0.31
Global cognition slope	−0.04 ± 0.04	−0.19 ± 0.09	−0.11 ± 0.09	<0.001
Aβ load	3.12 ± 3.77	5.93 ± 4.46	4.46 ± 4.50	<0.001
Tau density	2.97 ± 3.36	9.36 ± 9.15	5.98 ± 7.15	<0.001
Male, no. (%)	49 (34)	42 (27)	136 (32)	0.16
*APOE*‐ε4 carrier, no. (%)	27 (19)	60 (38)	109 (27)	<0.001

*Note*: Values are presented as mean ± standard deviation unless otherwise indicated. *P* values of comparison between cognitive normal controls and AD patients were derived from Welch two‐sample *t* test for continuous variables and Pearson chi‐squared test for categorical variables.

Abbreviations: Aβ, amyloid beta; AD, Alzheimer's disease; *APOE*, apolipoprotein E; PMI, *post mortem* interval.

### 2.2 snRNAseq

Specimens were collected on the availability of frozen pathologic materials from the DLPFC. The tissue specimens were collected by the Rush Alzheimer's Disease Center and processed at Columbia University Medical Center. Participants with a PMI < 41 hours and whole genome sequence data were selected to go through a series of rigorous quality control steps,^20^, ^24^, ^25^ which resulted in 424 participants. The participants had a median of 3824 sequenced nuclei. In the final data, there were eight major cell types, including astrocytes, vascular niche (referred to as “endothelial cells” or “endo” in our study), inhibitory neurons, microglia, oligodendrocytes, OPCs, and *CUX2*– and *CUX2*+ excitatory neurons. These eight major cell types were further subclustered into 95 cell subpopulations.^19^ For association and intercellular communication analysis we merged 19 subtypes within the vascular niche into eight larger subtypes, including endothelial cells, pericytes, smooth muscle cells, fibroblasts, arteriole, venule, erythrocytes, and immune cells. snRNAseq data can be downloaded from the AD Knowledge Portal (Accession Number: syn31512863). Additional filters applied before analyses included removing genes with expression in < 10% of all cells, removing cells with < 200 or > 20,000 total RNA Unique Molecular Identifiers (UMIs), or having > 5% mitochondrial mapped reads.

**RESEARCH IN CONTEXT**

1. **Systematic review**: We reviewed the literature using traditional sources (e.g., PubMed and Google Scholar). The role of the vascular endothelial growth factor (VEGF) in the pathogenesis of Alzheimer's disease (AD) has been recently described, including notable changes along the VEGFB/FLT1 signaling pathway. However, the cell type–specific role of the VEGFs in the pathogenesis of AD is not well characterized.
2. **Interpretation**: Our findings suggest that prefrontal cortical expression of *FLT1* and *FLT4* was associated with worse global cognitive function and cognition decline, and more AD neuropathology. These associations appear to be driven by endothelial and microglial cells. In contrast, *VEGFB* expression seems to have opposing effects on the amyloid burden in AD depending on the cell types, suggesting its more dynamic role in disease pathology.
3. **Future directions**: Our work highlights the cell type–specific effect of VEFG family genes on AD endophenotypes, but is limited to non‐Hispanic White, highly educated individuals and in dorsolateral prefrontal cortex tissue. Further investigation will be needed to explore a larger, more diverse cohort and other AD‐affected brain tissues.

### 2.3 Measurements of cognitive function

The measurement of global cognition[Table alz14419-tbl-0001] was derived from 17 different neuropsychological tests across five domains of cognition (semantic, episodic, and working memory; perceptual speed; and perceptual orientation). The *z* scores of all the available tests were averaged to create a global cognition composite. Further details on the development of this composite have been previously described.^26^ For cross‐sectional cognition function–related analysis, the global cognitive score at the last visit before death was used from 369 participants. Longitudinal cognitive trajectory was derived from a linear mixed effects model with global cognition as the outcome and the intercept and interval (years from the last visit) entered as fixed and random effects. The derived “cognition slope” was calculated as the sum of the random and fixed effects for each participant. Four hundred twenty‐three participants are included in this analysis.

### 2.4 Measurements of AD pathology

Measures of pathology were previously characterized in ROS/MAP.^21^, ^22^ Measured by immunohistochemistry at autopsy, Aβ load and tau tangle density were quantified as the average percent area occupied by Aβ (using antibodies specific to Aβ_42_) or tau (using antibodies specific to AT8 epitope of abnormally phosphorylated tau) across eight brain regions at autopsy: hippocampus, angular gyrus, and entorhinal, mid frontal, inferior temporal, calcarine, anterior cingulate, and superior frontal cortices. Then, values were transformed to approximate a normal distribution. Four hundred twenty‐one participants are included in Aβ load and tau density analysis.

### 2.5 Association and statistical analysis

Statistical analyses were completed using RStudio (R version 4.3.1) and code is available upon request from the corresponding author. Significance was set to a priori to *α *= 0.05. *P* values were first corrected for all *VEGF* predictors across AD‐associated outcomes and cell types (290 models for the eight major cell type analyses, 1,036 models for cell subpopulation analyses) using the false discovery rate (FDR) procedure. In addition, to provide context for these results, we also recalculated all models including all genes measured in each cell type for each outcome. Then we ranked all associations by *p* values and performed a correction for multiple comparisons within cell types using the Bonferroni procedure (please refer to Table S1 in supporting information for the number of models corrected per cell type and outcome). We report the rank of each VEGF family gene association and the transcriptome‐wide Bonferroni corrected *p* values to provide context for how strong each VEGF association is relative to the rest of the genome.

Negative binomial mixed models implemented by the Nebula R package^27^(v1.4.2) were used to analyze the association between each *VEGF* gene within each of the eight major cell types as well as within each of the 18 microglia, ten astrocytes, and eight vascular niche cell subtypes (as described in section 2.2) and AD diagnosis, global cognition longitudinally and at last visit before death, and Aβ load and paired helical filament tau tangle density at death. For analysis of diagnosis, 299 individuals were included: 142 normal cognition controls (cogdx = 1) and 157 AD dementia patients (cogdx = 4 or 5). All models covaried for age at death, sex, PMI, and the interval between the last cognitive visit and death in years (for longitudinal cognitive decline). One participant didn't have PMI data and hence was removed from association analysis. The gene count matrix input of the models was the UMI count data from the RNA assay normalized and scaled by the “sctransform” R package.^28^

### 2.6 VEGF‐mediated intercellular communication profiling

Intercellular communication patterns involving these ten VEGF genes among seven major cell types (*CUX2*+ and *CUX2*– were merged to excitatory neurons) and among eight vascular niche subtypes and the remaining six major cell types were analyzed using the “CellChat” R package (v1.0).^29^ Due to the lack of some of the evident ligand–receptor pairs involving these ten VEGF genes from the default CellChat database (CellChatDB), we manually updated CellChatDB by incorporating those curated additionally from the CellTalk DB (v1.0).^30^ All VEGF ligand–receptor pairs that were included in this study can be found in Table S2 in supporting information. We performed the rest of the analysis according to the default setting of CellChat, omitting projecting gene expression data onto the human protein–protein interaction network.

## 3 RESULTS

### 3.1 *VEGF* associations with cognition

Higher expression of *FLT1* in endothelial cells and microglia was associated with worse cognitive performance at last visit before death (endothelial–*FLT1* [logFC = −0.089, FDR = 0.009] and microglia–*FLT1* [logFC = −0.176, FDR = 0.009]) and faster cognitive decline (endothelial–*FLT1* [logFC = −0.771, FDR = 0.041] and microglia–*FLT1* [logFC = −1.964, *p* = 0.009]). Upon further investigation of the *FLT1* associations among microglia cell subtypes, it seems that Mic. 5 (surveilling microglia) and Mic. 6 (reacting microglia) subtypes were the ones that drove the microglia associations with worse cognitive performance at last visit before death (Mic.5–*FLT1* [logFC = −0.294, FDR = 0.007], Mic.6–*FLT1* [logFC = −0.310, FDR = 0.016]; Table S3 in supporting information). For vascular niche cells, the venule cell subpopulations seemed to be the main player that drove the *FLT1* association with worse cognitive performance at the last visit (logFC = −0.110, FDR = 0.052; Table S3). Additionally, higher expression of *VEGFB* in oligodendrocytes (logFC = −0.561, FDR = 0.019) was also associated with faster cognitive decline. Table 2 listed all statistically significant associations between ten VEGF gene expressions from eight major cell types and various AD outcomes tested together with their transcriptome‐wide Bonferroni corrected *p* values (the complete results can be found in Table S4 in supporting information).

**TABLE 2:** FDR significant cell type–specific VEGF associations with cognition and AD pathology.

Gene	Outcome	Cell type	logFC	SE	*p*‐value	FDR	Global rank
*FLT1*	Last cognition before death	Endothelial cells	−0.089	0.024	1.768E‐04	0.009	Top 0.44%
*FLT1*	Last cognition before death	Microglia	−0.176	0.046	1.492E‐04	0.009	Top 0.22%
*FLT1*	Cognition trajectory	Endothelial cells	−0.771	0.248	0.002	0.041	Top 1.99%
*FLT1*	Cognition trajectory	Microglia	−1.964	0.504	9.716E‐05	0.009	Top 0.34%
*VEGFB*	Cognition trajectory	Oligodendrocytes	−0.561	0.166	7.232E‐04	0.019	Top 2.46%
*FLT1*	Diagnosis	Endothelial cells	0.195^*^	0.055	4.232E‐04	0.014	Top 0.79%
*FLT1*	Diagnosis	Microglia	0.452^*^	0.113	6.378E‐05	0.009	Top 0.11%
*FLT1*	Aβ load	Microglia	0.053	0.011	2.700E‐06	0.001	Top 0.40%
*VEGFB*	Aβ load	Oligodendrocytes	0.014	0.004	1.428E‐04	0.009	Top 3.09%
*VEGFB*	Aβ load	Inhibitory neurons	−0.012	0.004	6.529E‐04	0.019	Top 8.69%
*FLT4*	Aβ load	Endothelial cells	0.027	0.008	3.579E‐04	0.013	Top 0.27%
*VEGFD*	Aβ load	Astrocytes	0.016	0.005	0.001	0.029	Top 3.96%
*NRP1*	Aβ load	Oligodendrocyte precursor cells	0.018	0.006	0.002	0.046	Top 4.72%
*NRP1*	Tau density	Astrocytes	−0.014	0.004	2.229E‐04	0.009	Top 1.62%

Abbreviations: Aβ, amyloid beta; AD, Alzheimer's disease; FDR, false discovery rate; SE, standard error; VEGF, vascular endothelial growth factor.

* logFC values of diagnosis are log2 fold changes of gene expression comparing AD to cognitively normal participants.

### 3.2 *VEGF* associations with AD pathology

Multiple cell types and *VEGF* genes were associated with Aβ load (Table 2). Higher microglia–*FLT1* (logFC = 0.053, FDR = 0.001), oligodendrocyte–*VEGFB* (logFC = 0.014, FDR = 0.009), endothelial–*FLT4* (logFC = 0.027, FDR = 0.013), astrocyte–*VEGFD* (logFC = 0.016, FDR = 0.029), and oligodendrocyte precursor cell–*NRP1* (logFC = 0.018, FDR = 0.046) were all associated with higher Aβ load. Most likely Mic. 8 (reacting microglia; logFC = 0.072, FDR = 0.004) and Mic. 3 (surveilling microglia; logFC = 0.048, FDR = 0.025) subpopulations drove the *FLT1*–Aβ association, while endothelial cells (logFC = 0.027, FDR = 0.075) and pericytes (logFC = 0.142, FDR = 0.075) probably drove the *FLT4*–Aβ association. In contrast, within inhibitory neurons, higher expression of *VEGFB* was associated with lower Aβ load (logFC = −0.012, FDR = 0.019). On the other hand, we only found one gene that associated with tau tangle density, which was higher astrocyte‐expressed *NRP1* associated with lower tau density (logFC = −0.014, FDR = 0.009, Table 2), which seemed to be driven by Ast. 3 (enhanced‐mitophagy/translation astrocytes; logFC = −0.011, FDR = 0.052) and Ast. 6 (logFC = −0.016, FDR = 0.095) cell subtypes.

To examine whether the contribution of microglia–*FLT1* and oligodendrocyte–*VEGFB* to more Aβ load influences the association of their expression with worse cognitive performance, we performed a competitive hierarchical analysis (“gene expression ∼ sex + age at death + PMI + Aβ + cognition [either cross‐sectional score at last visit or longitudinal trajectory]” using the same Nebula setting for other association analyses). We found that when covarying for Aβ, both gene associations with cognitive performance were attenuated ≈ 20% (by comparison of logFC values), but remained statistically significant for microglia–*FLT1* (*P* = 0.011 for both cross‐sectional and longitudinal cognitive performance models) and fell just below statistical significance in the case of oligodendrocyte–*VEGFB* (*P* = 0.065 for longitudinal cognitive performance model). Together our analysis indicated that the associations of microglia–*FLT1* and oligodendrocyte–*VEGFB* to cognitive performance are only partially explained by their association with Aβ load.

### 3.3 Differential expression of VEGFs in AD

We also tested for differences in VEGF expression between neuropathologically confirmed AD dementia cases and cognitively unimpaired. We observed that AD patients had higher expression levels of *FLT1* within microglia (logFC = 0.452, FDR = 0.009) and endothelial cells (Table 2, logFC = 0.195, FDR = 0.014).

### 3.4 VEGF‐associated intercellular communication profile

**FIGURE 2:**
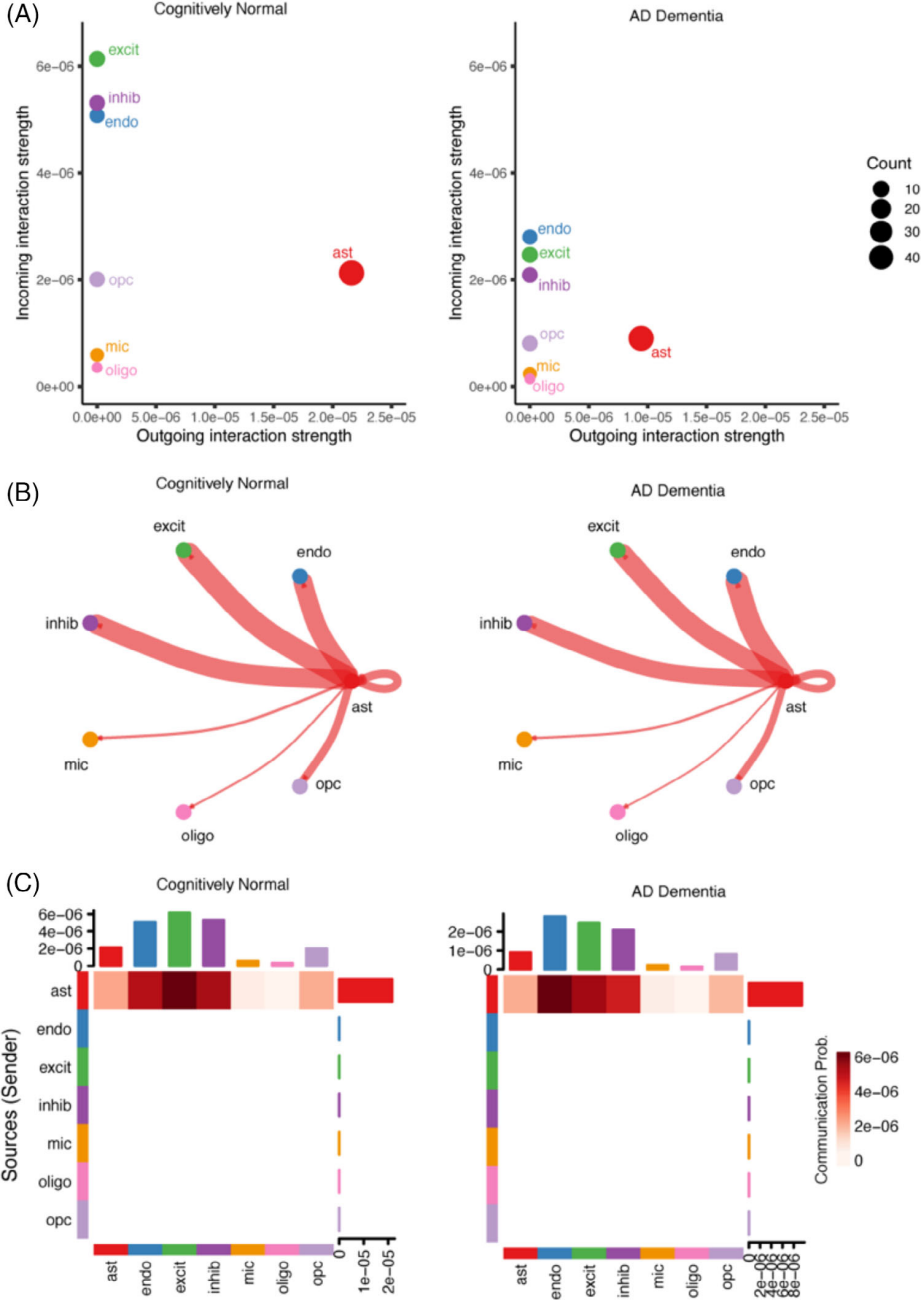
Ten VEGF‐associated signaling pathway profile in AD versus cognitively normal groups. A, Overall incoming and outgoing VEGF signaling strength comparison. B, Circle plot of overall VEGF information flow. C, Heatmap of overall VEGF information flow. AD, Alzheimer's disease; Ast, astrocytes; CUX2– Excit, all excitatory neurons that don't express *CUX2*; CUX2+ Excit, *CUX2*+ excitatory neurons; Endo, endothelial cells; Inhib, inhibitory neurons; Micro, microglia; Oligo, oligodendrocytes; OPC, oligodendrocyte precursor cells; VEGF, vascular endothelial growth factor.

Overall VEGF communication strength (calculated by summing communication probabilities of all curated VEGF pathway ligand–receptor (L–R) pairs among all cell types) (Table S5 in supporting information) was significantly reduced (Wilcoxon test; *P* < 0.05) in the AD group compared to the cognitively normal group, and the reduction was observed for both incoming and outgoing VEGF signaling strength (calculated by in‐degree and out‐degree network centrality scores, respectively) among cells (Figure 2A). *VEGFA* was the most abundantly expressed VEGF ligand among the investigated, primarily in astrocytes; hence, it was no surprise to see many of the significant interactions (L–R pairs) for VEGF pathways were with *VEGFA* for both AD and cognitively normal groups. As a result, overall VEGF information flow among these brain cells was from astrocytes to other cell types including themselves (Figure 2B). But interestingly, the top receiver cell type of VEGF communications for the cognitively normal group was excitatory neurons, whereas for the AD group, it was endothelial cells (Figure 2C). The underlying cause of this difference lay in the significant change in the relative contribution of top L–R pairs to the overall VEGF communications when comparing these two diagnosis groups. Specifically, “VEGFA–GRIN2B” was 37.8% in AD versus 39.5% in the cognitively normal group, “VEGFA–SIRPA” was 10.2% versus 10.7% (AD vs. cognitive normal), and “VEGFA–EGFR” was 10.2% versus 9.5% (AD vs. cognitive normal). But probably the most important change is for “VEGFA–FLT1,” which contributed 13.8% of all VEGF signals in AD dementia patients compared to 9.5% in cognitively normal individuals. VEGFA–FLT1 signal is from astrocytes to endothelial cells exclusively, and it showed a comparable communication probability between cells in AD and cognitively normal participants (adjusted probability of 0.073 vs. 0.076), which can be seen as an outsider considering the overall reduction of VEGF signals in the AD group (Table S5 presents the complete result). The significantly elevated *FLT1* expression in endothelial cells from AD patients might have facilitated this change.

## 4 DISCUSSION

In this study, we provided the most comprehensive exploration of cell‐specific effects of the VEGF family in the context of AD. Our results build on previous evidence from bulk tissue at the protein and transcriptomic level that *VEGFB, FLT1, FLT4*, and *NRP1* relate to the clinical progression and neuropathology of AD.^16^, ^18^ The single‐cell resolution here builds on our early single nucleus data^18^ to provide the clearest picture of the important and distinct roles the VEGF family members play in various brain cell types. Our results particularly highlight the diverse associations for VEGF family members across endothelial cells, microglial cells, astrocytes, oligodendrocytes, and neurons. While *FLT1* showed strong upregulation in the AD brain in endothelial cells as might be expected, we also observed strong upregulation of *FLT1* in microglial cells. Yet, regardless of the upregulation of *FLT1*, our intercellular communication analyses suggest that FLT1‐associated canonical VEGF signaling pathways themselves are in fact comparable in the AD brain to normal brain. In contrast to the consistent effects observed for *FLT1*, we observed opposing directions of effect on Aβ load for *VEGFB* expression in oligodendrocytes versus inhibitory neurons, whereby higher levels of oligodendrocyte *VEGFB* related to higher pathology, while higher levels of neuronal *VEGFB* related to lower pathology. Together, these results suggest that in addition to the classic vascular signaling pathway underlying the associations between VEGF family members and AD, there may also be immune (e.g., microglial *FLT1*) and neuronal (e.g., neuronal *VEGFB*) signaling cascades that contribute to AD pathogenesis.

The central role of VEGF signaling is angiogenesis under both health and disease conditions.^31^, ^32^ Pathological angiogenesis is a hallmark of AD, which is believed to both drive and respond to multiple AD pathologies driving hypoxia, oxidative stress, inflammation, and Aβ accumulation.^33^ Indeed, previous work has identified a marked upregulation of angiogenic factors and mediators in the AD brain.^34^, ^35^ In the present results, the upregulation of *FLT1* in endothelial cells among AD patients could reflect a repair response. The increase in vascularization and blood flow could be a response to AD neuropathology similar to the response that has been indicated in other neurodegenerative and neuropsychiatric diseases such as amyotrophic lateral sclerosis^36^ and schizophrenia.^37^ On the other hand, the elevated endothelial *FLT1* could drive AD neuropathology. For example, it has been shown that VEGF‐induced pathological angiogenesis can facilitate Aβ generation.^38^ VEGF‐induced pathological angiogenesis can also worsen AD pathology by disrupting the permeability of the blood–brain barrier (BBB).^39^, ^40^ BBB is formed by the physical interaction of multiple brain cell types, with a common structure consisting of blood capillaries formed by endothelial cells, pericytes, and the astrocytic end feet.^41^ As a natural protective barrier of the central nervous system (CNS), BBB homeostasis is strictly regulated, disruption of which can lead to many neurological conditions including AD.^42^, ^43^ Our intercellular communication profile showed that signals communicated among these ten VEGF genes were predominantly from astrocytes to endothelial cells, hinting at the canonical angiogenesis pathway at the BBB as a primary driver here. In fact, evidence is already emerging that demonstrates CNS angiogenesis can be regulated through VEGF‐dependent signaling at the BBB.^44^ Characterizing the interplay between VEGF family genes and BBB integrity in the context of AD may provide more insight into the mechanistic pathways underlying the associations observed here.

Notably, previous work also observed the downregulation of genes for effectors of angiogenic and trophic signaling including VEGFA‐VEGFR2 (e.g., VEGFA‐KDR).^35^ This is consistent with our overall observation of a significant reduction of VEGF signaling in AD dementia patients compared to cognitively normal controls. However, our dataset did not show any differential expression patterns of *VEGFA* or *KDR* between diagnostic groups. Given that *KDR* expression in endothelial cells is much lower than *FLT1* (data not presented), it is perhaps unsurprising that we were not able to detect any meaningful alterations in the VEGFA/KDR signaling pair. That said, more focused isolation of vascular cells may provide additional sensitivity to subtle alterations in the VEGFA/KDR axis as reported elsewhere.

Our results also suggest the roles of some of the VEGF family genes in regulating the immune response, including the upregulation of microglial *FLT1* in the AD group. Increased microglia function in AD has been observed by many^45^, ^46^, ^47^, ^48^, ^49^ in the presence of Aβ peptides and facilitates an inflammatory response.^50^ FLT1 was believed to be one of the 20 or so proteins that has been found to be enriched in Aβ plaques,^51^ and has recently been shown to interact with VCAM1 and plays a role in VCAM1‐dependent chemotaxis of microglia, which enhances microglia clearance of Aβ.^52^ Studies have also shown the critical role that FLT1 plays in microglial proliferation and monocyte–macrophage migration.^53^, ^54^ With all these under consideration, the elevation of microglial *FLT1* in AD should not be a surprise. The primary ligand for FLT1 is VEGFB, so it is possible that the *VEGFB* association in oligodendrocytes may reflect the upregulation of this same pathway. It is well known that oligodendrocytes and microglia work together during the regulation of myelination, and this is particularly noticeable during neurodegenerative conditions.^55^ Future work confirming these cell type–specific transcriptomic effects at the protein level is necessary.

One fascinating discovery in the present results is that higher expression of *VEGFB* within inhibitory neurons was associated with lower Aβ load, which was the only protective effect of any VEGF we tested in this dataset. This result is consistent with the previously reported evidence of the benefits of *VEGFB* expression on neuronal survival and nerve regeneration.^56^, ^57^, ^58^, ^59^ Perhaps in the early stage of AD when Aβ load is low, inhibitory neurons manage to inhibit apoptosis and promote neurogenesis through upregulating *VEGFB*. Upregulation of *VEGFB* could be one of the molecular mechanisms that enable inhibitory neurons to be more resistant to AD neuropathology compared to excitatory neurons.^60^ Interestingly, a study of APP/PS1 amyloidopathy mice did show an increase of inhibitory synapses in the hippocampus before pathology developed and then decreased at 12 months of age when robust AD pathology was present.^61^ Hence, the protective effect of *VEGFB* from inhibitory neurons to the development of AD pathology could function in a time‐sensitive matter. One also cannot rule out the possibility that increased *VEGFB* expression within inhibitory neurons could help with the clearance of Aβ.

We also observed higher expression of *FLT4* within endothelial cells related to higher Aβ load. The primary ligand for FLT4 is VEGFD and we also observed an association between elevated *VEGFD* in oligodendrocytes and Aβ load. In contrast to the other VEGF receptors, the FLT4*/*VEGFD signaling axis is primarily involved in lymphangiogenesis. Certainly, meningeal lymphatics have been implicated in Aβ clearance through a complex interplay with the immune system,^62^ so it is quite possible that lymphatics underlie the observed *VEGFD* and *FLT4* associations with Aβ. In previous work, we identified an association between higher expression of *FLT4* in the DLPFC and worse neuropathology,^16^ but also identified higher expression of *FLT4* in the caudate nucleus associated with lower Aβ and tau levels.^18^ Others have also observed these flips in direction with associations depending on the brain region.^63^ Combined, these results suggest that tissue and cell type are important considerations when looking at the role of *FLT4* in the brain within the context of AD.

Finally, we observed a cell type–specific effect of *NRP1*. Higher expression of *NRP1* in astrocytes was associated with lower levels of neurofibrillary tangles, but higher expression of *NRP1* within oligodendrocyte precursor cells was associated with higher Aβ load. Other work from our group has also observed differing roles of *NRP1* when pertaining to AD neuropathology.^18^ However, this is the first time we have observed statistically significant associations within specific cell types. All the above results further highlight the complexity of the VEGF family and their role in neurodegeneration. To fully understand the clinical potential of *VEGF* as a biomarker and therapeutic target, an in‐depth understanding of each of the tissue and cell types is necessary.

This study has many strengths. ROS/MAP is a well‐characterized cohort with longitudinal cognitive data and comprehensive neuropathology data with multiple cell‐type measures of *VEGF* expression. Additionally, this study reports novel discoveries and replication of the effects of VEGF family members on AD phenotypes including pathology and cognition at the transcriptomic level within a large sample size. Because of the large sample size and cell type–specific expression measurements, we were able to provide more comprehensive data and hypotheses about cellular context.

Despite these strengths, this study also has limitations. ROS/MAP is enriched for non‐Hispanic White, highly educated individuals, which limits the generalizability to more representative populations. Further, while being able to examine cell type–specific effects in a large sample size, we are limited to the DLPFC tissue.

In summary, this study provides an extensive characterization within a large sample size of the *VEGF* signaling family members in the context of cognitive aging and AD. Our results provide evidence that further supports the role *VEGFB*, *VEGFD*, *FLT1*, *FLT4*, and *NRP1* play inside and outside of the classic VEGF signaling axis in AD, with support for both an endothelial and microglial signaling cascade that could underlie these well‐established associations. For the first time, we provide evidence that *VEGFB* expression in inhibitory neurons may relate to a protective effect against AD neuropathology as has been hypothesized previously and observed in models of Parkinson's disease. These results provide compelling evidence that *VEGFB* and *FLT1* may have potential as therapeutic targets in AD, but there remains a need to confirm these single nucleus transcriptomic results at the protein level and establish causality through experimental approaches.

## AUTHOR CONTRIBUTIONS

Timothy J. Hohman and Yiyang Wu designed the project. Yiyang Wu conducted all the analyses and analyzed the results. Yiyang Wu, Timothy J. Hohman, and Julia B. Libby wrote the manuscript. Logan contributed to data analysis, interpretation, and critical revision. Philip L. De Jager and Vilas Menon contributed to data generation, data analysis, funding, and critical revision. Julie A. Schneider and David A. Bennett contributed to data generation, funding, and critical revision. All authors read and approved the final manuscript.

## CONFLICT OF INTEREST STATEMENT

T.J.H. is a deputy editor for *Alzheimer's & Dementia: Translational Research and Clinical Intervention* and on the scientific advisory board for Vivid Genomics. All other co‐authors declare no conflict of interest. Author disclosures are available in the supporting information.

## CONSENT STATEMENT

Both ROS and MAP studies were approved by an institutional review board (IRB) of Rush University Medical Center. All participants signed informed and repository consents and an Anatomic Gift Act. Secondary analyses of this extant data were approved by the Vanderbilt University Medical Center IRB.

## Supporting information



Supporting Information

Supporting Information

## Data Availability

ROS/MAP data can be accessed online at the Rush Alzheimer's Disease Center Resource Sharing Hub (https://www.radc.rush.edu/), as well as on the Accelerating Medicines Partnership‐Alzheimer's Disease (AMP‐AD) Knowledge Portal (syn3219045).
